# Quality of Life Is Related to Social Support in Elderly Osteoporosis Patients in a Chinese Population

**DOI:** 10.1371/journal.pone.0127849

**Published:** 2015-06-10

**Authors:** Lina Ma, Yun Li, Jieyu Wang, Hong Zhu, Wei Yang, Ruojin Cao, Yuying Qian, Ming Feng

**Affiliations:** Department of Geriatrics, Xuan Wu Hospital, Capital Medical University, Beijing, 100053, China; University of Utah, UNITED STATES

## Abstract

**Objective:**

To explore the association between quality of life and social support in elderly osteoporosis patients in a Chinese population.

**Methods:**

A total of 214 elderly patients who underwent bone mineral density screening were divided into two groups: elderly patients with primary osteoporosis (case group, n = 112) and normal elderly patients (control group, n = 102). Quality of life and social support were compared between the two groups.

**Results:**

Quality of life and social support were significantly different between the case and control groups. The physical function, role-physical, bodily pain, general health, vitality, social-functioning, role-emotional and mental health scores in case group were significantly lower than those in the control group (P < 0.01). The objective support, subjective support, utilization of support, and total scores in case group were significantly lower than those in the control group (P < 0.01). Quality of life and social support were positively correlated in the case group (*r* = 0.672, P < 0.01).

**Conclusion:**

Quality of life and social support in elderly patients with osteoporosis in China were poorer than in elderly patients without osteoporosis and were positively correlated. Our findings indicate that increased efforts to improve the social support and quality of life in elderly osteoporosis patients are urgently needed in China. Further longitudinal studies should be conducted to provide more clinical evidence to determine causative factors for the observed association between risk factors and outcomes.

## Introduction

Estimated future demographic changes in China will result in significant social and economic challenges in the daily lives and care of elderly people, including maintenance of quality of life (QOL). Osteoporosis (OP), which is characterized by low bone mass, bone microstructure damage, bone fragility, and risk of fractures, is one of the most common diseases in the elderly, with annual increases in the prevalence of osteoporotic fractures with the trend of improved population aging[1,2]. The incidence of OP in the elderly population is as high as 56%, and women represent 60–70% of elderly people with OP in China [3–5]. Osteoporotic fracture is associated with high rates of disability and mortality in elderly patients[6,7]. Patients with OP have high disability and mortality rate, and the associated psychological burden and mental pain can affect QOL [8,9]. Despite increasing evidence showing worse QOL in OP patients, little is known regarding interventions improving QOL, particularly in elderly patients. Recently, a study in China has found over two years of zoledronic acid treatment in women with postmenopausal OP can improve bone mineral density, and can help improve QOL[10].

Social support is defined as the amount of affection, companionship, and care from family members, friends, and other individuals[11,12]. Brennan SL investigated the relationship between socioeconomic status and reported perceptions of QOL in a cross-sectional population-based analysis of a representative sample of Australian men, and found that men from lower and upper socioeconomic status groups had lower QOL compared to their counterparts in the mid socioeconomic status group[13]. Low social support and low socioeconomic status significantly increased the odds of depressive symptoms[14]. Better social support is reported to be associated with improved QOL, and individuals with OP who have lower pain and more exercise are considered having better QOL[15]. Social support predicts QOL above and beyond disease activity, demographic factors and social integration in patients with rheumatoid arthritis[16], and measurement of QOL plays an important role in the clinical evaluation of OP and comparison of different therapeutic measures in clinical trials[17,18]. Social support was also found to partially mediate loneliness and depression in elderly people[19], and it has beneficial effects on subjective well-being of older adults among domains of enjoyment, morale, depression and loneliness [20,21], which are closely relative to QOL. Pediatric research has found that family social support could predict healthy lifestyle behaviors for the prevention of OP in a population with 354 girls, aged 8–11 years [22]. A study containing 112 female patients with osteoporosis and 112 normal controls in community in China showed the incidence of depression in OP patients was higher, the QOL was worse, and the score of objective support was lower[23]. Nevertheless, to our knowledge, there is no research on social support and QOL in elderly patients with OP, and our study is the first to concentrate on this. The hypothesis of this research was that social support and QOL in elderly patients with OP in China were worse than normal controls, and were positively correlated.

## Materials and Methods

### Participants

Elderly patients (average age, 69.27 ± 9.21 years) with primary OP (n = 112; 81 men, 31 women) and normal elderly patients (average age, 68.26 ± 8.62 years) (n = 102; 74 men, 28 women) were recruited from the Department of Geriatrics, Xuan Wu Hospital, Capital Medical University between August 2008 and September 2012. Exclusion criteria included secondary OP; diabetes; cancer; recent acute infection; severe cardiac, liver, or kidney dysfunction; cerebrovascular disease; severe Parkinson's disease; depression or anxiety; dementia; and trauma or operation in the previous six months.

All of the participants underwent a standardized clinical assessment, which included a medical history, physical examination, completion of the Short Form-36 (SF-36) questionnaire, completion of the Social Support Rating Scale (SSRS), and BMD measurement. Informed consent was obtained from all participants prior to participation. This clinical investigation was approved by the Ethics Committee of Xuanwu Hospital, Capital Medical University, China. Participants provide their written informed consent to participate in this study. The participant consent was recorded in a file and the ethics committee approved this consent procedure.

### Data collection

In this prospective study, a comprehensive questionnaire was completed by trained investigators. The following basic demographic and clinical data were retrospectively obtained from the medical charts: sex, age, duration of admission, diagnosis, and medication history.

Venous blood was collected from the patients who were admitted to the hospital early in the morning following an overnight fast. Serum total cholesterol, triglycerides, C-reactive protein, high-density lipoprotein cholesterol, low-density lipoprotein cholesterol, creatinine, and fasting plasma glucose were determined.

The SF-36 scale was used to assess QOL. The scale consists of 36 items within 8 scales that assess the following general health concepts: physical function (PF), role-physical (RP), bodily pain (BP), general health (GH), vitality (VT), social-functioning (SF), role-emotional (RE), and mental health (MH). Each of the 8 scales has a lowest possible score of 36 and a highest possible score of 150. Higher scores indicate better QOL[24]. The total score and each factor score were calculated.

The SSRS was used to evaluate social support. The SSRS contains a total of 10 items within three dimensions: objective support (3 items), subjective support (4 items), and the utilization of support (3 items). Objective social support refers to actual or visible social support, including material direct assistance and social relationship network. Subjective social support refers to the experience or emotional social support, namely the individual feels understood and respected by society. Utilization of support refers to the conscious or unconscious use their social support system to cope with challenges or stress. Higher SRSS scores indicate greater social support[25].

### Bone density

All subjects underwent a BMD measurement by a trained technician in a separate room at the hospital using dual energy X-ray absorptiometry (DXA) (GE, Madison, WI, USA) for the lumbar (L1-L4) and femoral neck regions as well as the whole body. DXA is considered the gold standard method for the diagnosis of OP because it is rapid, safe, and accurate[26]. Absolute values of BMD are expressed as T-scores (standard deviation from the normal reference population) and Z-scores (standard deviation from the sex- and age-matched population). OP was diagnosed according to the WHO criteria[27]: osteopenia (bone mass loss) is 1–2.4 standard deviations below the mean BMD of healthy adults of the same sex and race; OP is ≥2.5 standard deviations below the mean BMD of healthy adults of the same sex and race[28]. Spine and hip radiography were conducted to identify a vertebral or hip compression fracture. Quality control for the assessment was carried out in strict accordance with the consensus of the International Society for Clinical Densitometry.

### Statistical analysis

Data are expressed as mean ± SEM. Unpaired *t*-tests were conducted for data that were normally distributed, and rank sum tests were conducted for data that were not distributed normally. Pearson correlation analysis was conducted to evaluate the correlation between QOL and social support in both the case and control groups. Moderated regression analysis using dummy coding was conducted. Differences were considered statistically significant and very significant at P < 0.05 and P < 0.01, respectively. SPSS version 12.0 for Windows (SPSS Inc., Chicago, IL, USA) was used for all statistical analyses.

## Results

### Comparison of common factors between the two groups

There were no significant differences between the two groups in age, sex, marital status, the levels of serum total cholesterol, triglycerides, C-reactive protein, high-density lipoprotein cholesterol, low-density lipoprotein cholesterol, creatinine, fasting plasma glucose or body mass index (all P>0.05) (Table 1).

**Table 1:** Comparison of common factors between the two groups.

Groups	Number	Age (Years old)	Sex (Male,%)	Marital status (Married,%)	TC (mmol/L)	TG (mmol/L)	HDL-C (mmol/L)	LDL-C (mmol/L)	Cr (mmol/L)	FPG (mmol/L)	BMI (kg/m^2^)
Case group	112	69.27±9.21	72.32	98.04	4.7±0.4	1.7±0.2	2.2±0.4	2.5±0.4	75.2±16.0	5.6±0.4	22.5±4.1
Control group	102	68.26±8.62	72.55	98.21	4.6±0.3	1.8±0.2	2.1±0.3	2.4±0.2	74.0±17.2	5.8±0.7	23.0±3.9

Compared to the control group, all P > 0.05. TC: total cholesterol; TG: triglyceride; HDL-C: high density lipoprotein cholesterol; LDL-C: low density lipoprotein cholesterol; CRP: C-reactive protein; Cr: creatinine; FPG: fasting plasma glucose; BMI: body mass index.

### Comparison of quality of life scores between the two groups

**Fig 1:**
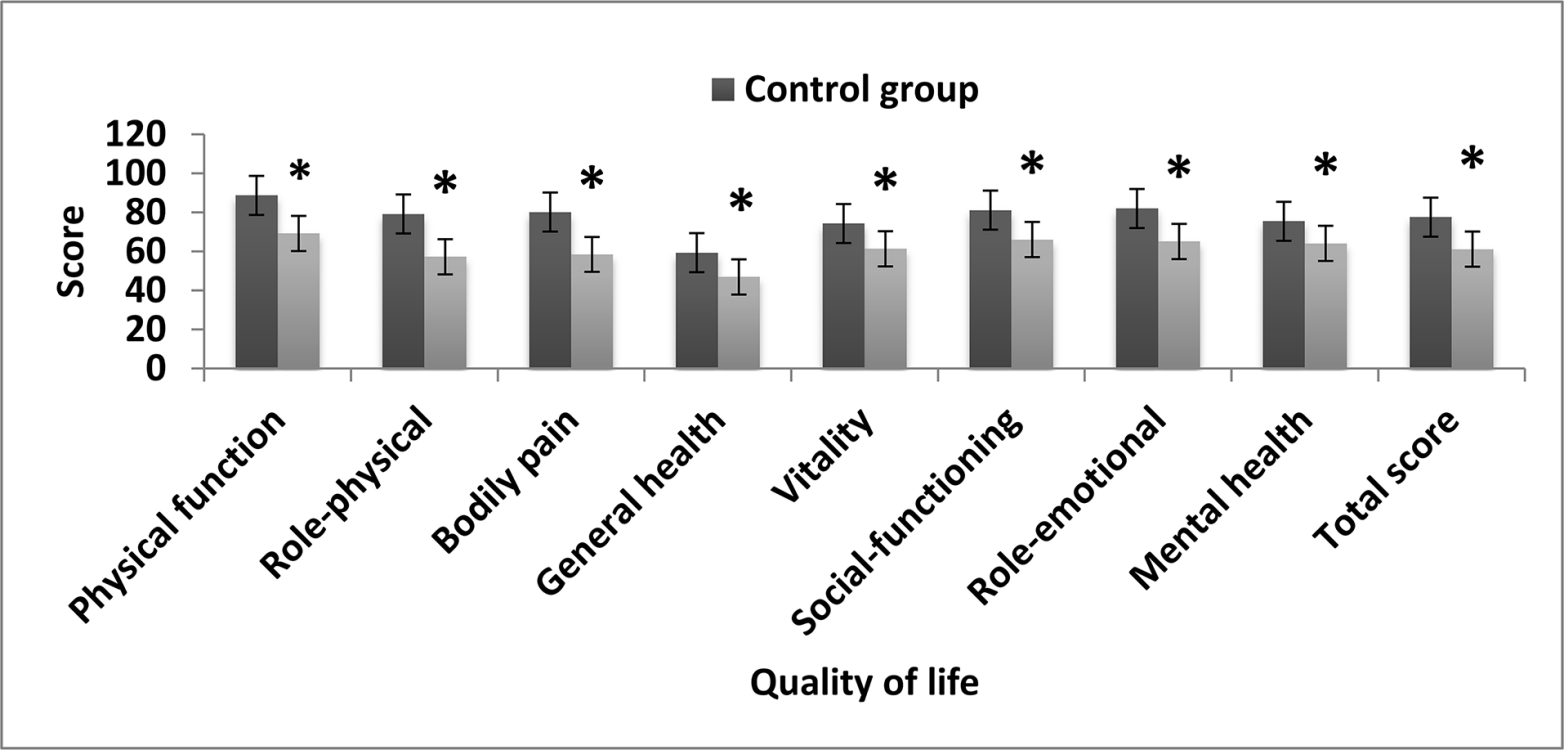
Comparison of SF-36 scores between the case group of elderly patients with osteoporosis (n = 112) and control group (n = 102). Values are reported as mean ± SEM. *Significantly different from the control group (P < 0.01).

The SF-36 showed the following scores in the case and control groups, respectively: PF, 69.2 ± 20.2 vs. 88.7 ± 17.1; RP, 57.2 ± 22.7 vs. 79.1 ± 22.6; BP, 58.4 ± 18.9 vs. 80.1 ± 20.4; GH, 46.9 ± 18.9 vs. 59.3 ± 18.0; VT, 61.3 ± 20.1 vs. 74.3 ± 20.2; SF, 66.0 ± 25.3 vs. 81.1 ± 30.0; RE, 65.1 ± 31.1 vs. 82.0 ± 33.5; MH, 64.0 ± 20.2 vs. 75.4 ± 22.0; and total, 61.1 ± 20.0 vs. 77.5 ± 23.4. The PF, RP, BP, GH, VT, SF, RE, MH and total scores in the OP group were significantly lower than those in the control group (P < 0.01) (Fig 1).

### Comparison of Social Support Rating Scale scores between the two groups

**Fig 2:**
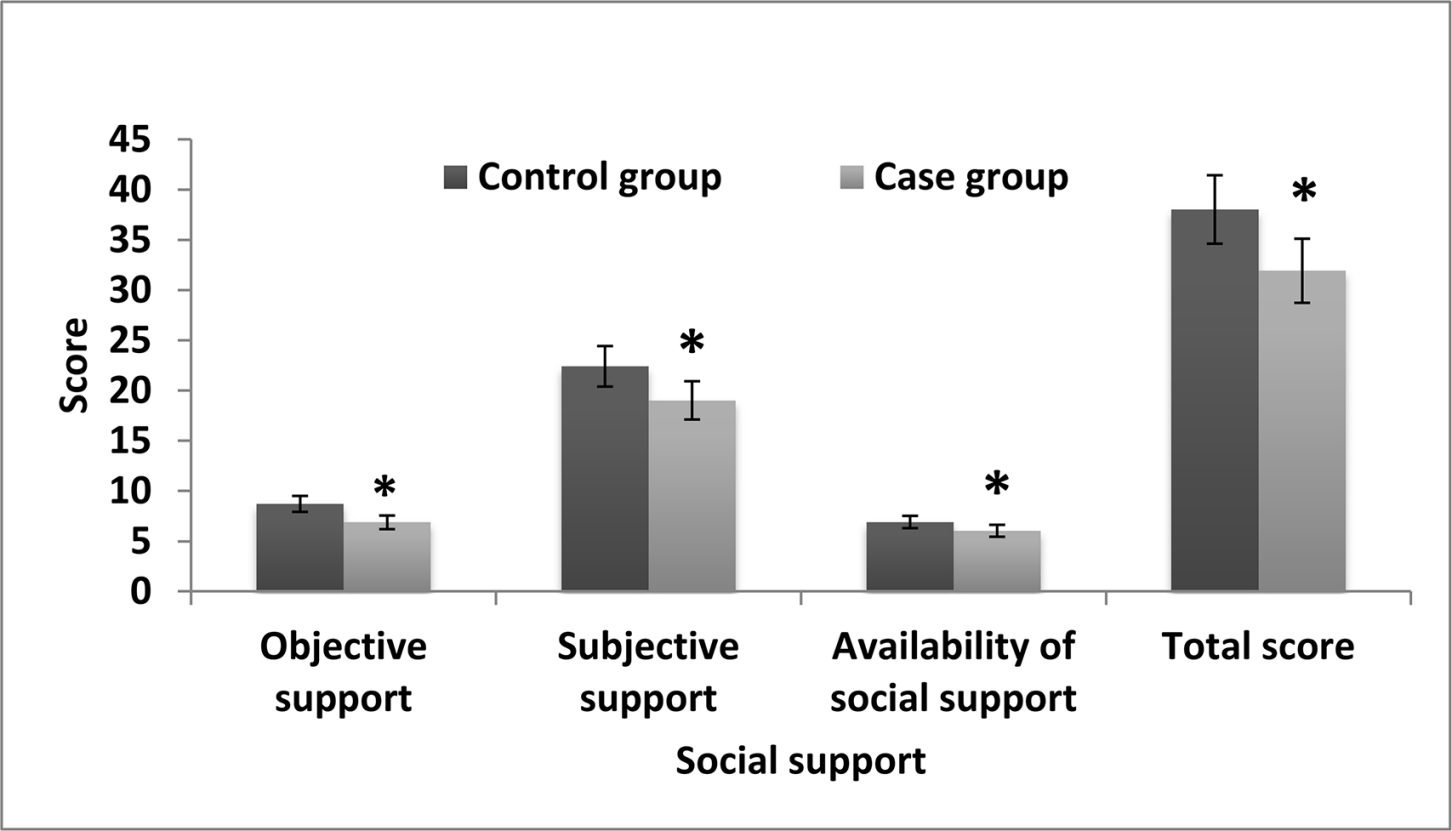
Comparison of Social Support Rating Scale scores between the case group of elderly patients with osteoporosis (n = 112) and control group (n = 102). Values are reported as mean ± SEM. *Significantly different from the control group (P < 0.01)

The Social Support Rating Scale resulted in the following scores for social support in the case and control groups, respectively: objective support, 6.88 ± 2.07 vs. 8.71 ± 2.66; subjective support, 19.02 ± 3.20 vs. 22.41 ± 4.10; utilization of support, 6.03 ± 1.25 vs. 6.91 ± 1.67; and total, 31.93 ± 7.83 vs. 38.03 ± 8.91. The objective support, subjective support, utilization of support, and total scores in the OP group were significantly lower than those in the control group (P < 0.01) (Fig 2, S1 Table.).

### Correlation between quality of life and social support in elderly osteoporosis patients

Social support was positively related to QOL in case group (*r* = 0.672, P < 0.01), while there was no correlation between social support and QOL in control group (*r* = 0.116, P = 0.244). Moderated regression analysis using dummy coding showed that there was no difference between the case and control group correlation (B = 0.245, t = 0.794, P = 0.428).

## Discussion

The hypothesis of this research was that social support and QOL in elderly patients with OP in China were worse than normal controls, and social support and QOL in case group were positively correlated. Our results showed a significant statistical difference in QOL and social support scores between the two groups, and this significant difference was observed in all dimensions of QOL and social support. Our study showed that QOL was significantly poorer in patients with OP than in the control group. In addition, patients with OP likely required higher levels of social support than they were reporting in the current study, and social support was positively related with QOL in the elderly patients with OP. This indicates that maybe we can provide adequate social support to improve the quality of life in elderly osteoporosis patients. To our knowledge, our study is the first to concentrate on social support and QOL in elderly patients with OP. Our results conform to the results of the study conducted by Pitsilka DA in patients with rheumatoid arthritis[16], but another study in China has found that objective support in patients with OP was lower than that of a control group, while there were no difference in other dimensions of social support between case group and control group[19], the discrepancy may be lie in the difference of study population, our study population contains both male and female elderly OP patients aged >60 years, while Wang’s study population contains female patients with postmenopausal OP aged >45 years.

With advances in the study of QOL, greater attention has been given to the QOL of patients with OP instead of just focusing on decreasing the rate of osteoporotic fractures. QOL can provide a comprehensive basis for reasonable allocation of health resources for treatment or intervention and decisions regarding screening in patients with chronic illnesses [29]. Decreased QOL in elderly patients with OP not only related to decreased labor capacity and psychological factors but also related to cardiovascular diseases[19,30,31]. Osteoporotic fracture is a major cause of morbidity and is negatively associated with QOL [32–34]. Patients with OP also have poorer social support, which refers to the help and support of both spiritual and material needs from family, relatives, friends, colleagues, and other individuals and organizations, as well as the individual's degree of social support utilization[12]. Li H has found that friend support rather than family support could enhance the positive effect of social support [35]. Social networks are important for the perception of support[36]. Social support is the interdependent relationship between individuals or between the individual and group; this relationship can show the ability to cope with short-term challenges, stress, and social relationship deprivation[37]. Intergeneration social support, self-esteem, and loneliness have been significantly correlated with subjective wellbeing[38]. Social support could moderate the association between stress and depression and was found to have effects on depressive and anxiety symptoms[39,40]. High social support also has a protective effect among individuals with low impulsivity[41].

Our study demonstrates social support and QOL were worse in elderly patients with OP in China and they were positively correlated. There are various reasons for this. Firstly, the living situation of the elderly in China is different from that of western countries [42]. Most of the elderly in China are retired at home; some have questioned if the loss of social sense, rising prices, and change in roles have caused a decline in the standard of living[43]. Secondly, familial respect and care, spiritual support, and material support are particularly important for the elderly in China, which can consciously encourage the elderly to increase availability of and initiate social support[44]. The elderly might increase their utilization of social support if they are encouraged to spend more time with family and friends, actively participate in collective activities, and actively talk to family members or friends when encountering troubles or are confused with something[45]. Moreover, increased socialization is advantageous for emotional health. Previous studies have found patients with low levels of stress receive more total and subjective social support than patients with high levels of stress, which reflects the buffer that social support has on mental stress and the protective effect on mental health[46,47]. Therefore, the social support system may contribute to disease rehabilitation and improvement of QOL [48].

Social support has been shown to be equivalent to many classic risk factors for prognosis of some diseases; therefore, it can be used both as a tool for risk stratification and potential target for interventions to improve outcomes[49,50]. Previous study has found health education interventions can improve the physiological, psychological, and social function aspects of QOL[51]. We have found QOL and social support in elderly patients with OP were significantly lower than those in normal elderly individuals, and social support was positively related to QOL in the elderly OP group, while there was no correlation between social support and QOL in control group, which indicates that increased efforts to improve the social support and quality of life in elderly OP patients are urgently needed in China. Therefore, we should strengthen the social support network for elderly OP patients, by conducting health education, organizing patient clubs, and improving community awareness, with the aim of improving QOL and realizing a healthy aging society[52,53]. Further longitudinal studies also should be conducted to provide more clinical evidence for the relationship of social support and QOL in elderly OP patients.

## Conclusion

The results of the present study show that QOL and social support in elderly patients with OP were poorer and positively correlated in elderly OP patients in China. These findings indicate that increased efforts to improve the social support and quality of life in elderly OP patients are urgently needed in China.

## Limitations

Participation in this study was restricted to patients living in Beijing. Hence, our results may not be representative of the overall Chinese population. Moreover, the cross-sectional study design cannot determine causative factors for the observed association between risk factors and outcomes; therefore, follow-up studies should be conducted in the future.

## Supporting Information

S1 Table The Social Support Rating Scale scores between the case group of elderly patients with osteoporosis (n = 112) and control group (n = 102) (PDF). (PDF)
