# Supplementary material for: Quality of Life Is Related to Social Support in Elderly Osteoporosis Patients in a Chinese Population
Source: PLoS One. 2015 Jun 10;10(6):e0127849. doi: 10.1371/journal.pone.0127849 (PMC4465171; doi:10.1371/journal.pone.0127849)
Supplement: S1 Table — (PDF) [file pone.0127849.s001.pdf]

S1 Table. The Social Support Rating Scale scores between the two groups

Group	Objective support	Subjective support	Availability of social support	Total score
Control group	8.71±0.80	22.41±2.05	6.91±0.59	38.03±3.42
Case group	6.88±0.61	19.02±1.72	6.03±0.55	31.93±2.88
